# Case report: a case of masked subclavian artery stenosis in a haemodialysis patient

**DOI:** 10.1093/ehjcr/ytae590

**Published:** 2024-11-02

**Authors:** Ayako Tezuka, Masatake Kobayashi, Ryosuke Ito, Naotaka Murata, Kazuhiro Satomi

**Affiliations:** Department of Cardiology, Tokyo Medical University, 6-7-1 Nishishinjyuku, Shinjyuku-ku, 160-0023 Tokyo, Japan; Department of Cardiology, Tokyo Medical University, 6-7-1 Nishishinjyuku, Shinjyuku-ku, 160-0023 Tokyo, Japan; Department of Cardiology, Tokyo Medical University, 6-7-1 Nishishinjyuku, Shinjyuku-ku, 160-0023 Tokyo, Japan; Department of Cardiology, Tokyo Medical University, 6-7-1 Nishishinjyuku, Shinjyuku-ku, 160-0023 Tokyo, Japan; Department of Cardiology, Tokyo Medical University, 6-7-1 Nishishinjyuku, Shinjyuku-ku, 160-0023 Tokyo, Japan

**Keywords:** Subclavian artery stenosis, Endovascular treatment, Haemodialysis, Lower extremity artery disease, Case report

## Abstract

**Background:**

Subclavian artery stenosis is generally screened by a left–right brachial systolic blood pressure difference. However, subclavian artery stenoses are often underdiagnosed due to marginally identified symptoms. In dialysis patients, a relative or absolute contradiction of measuring blood pressure in shunt brachial artery may further limit the disease screening.

**Case summary:**

A 77-year-old female requiring dialysis presented with a suspected acute coronary syndrome complicated by cardiogenic shock. Five months before presentation, the patient was increasingly given inotropic drugs and had often chest discomfort during dialysis. An emergency coronary angiogram of the right coronary artery revealed 99% stenosis with hypoplasia. During catheterization, angiography of the aortic arch showed subtotal occlusion of the left subclavian artery. After revascularization, patients did not suffer from low blood pressure during haemodialysis.

**Discussion:**

Dialysis patients may have high perceived risk of subclavian artery stenosis. However, limitation of measuring blood pressure in shunt artery may enhance its underdiagnosis. Our case highlights the importance of screening for subclavian artery stenosis in patients undergoing dialysis.

**Learning points**

- To appreciate the challenge to diagnose subclavian artery stenosis in haemodialysis patients.
- Subclavian artery stenosis is one of the causes of haemodialysis-associated hypotension.
- Endovascular therapy for unilateral subclavian artery stenosis in the non-fistula arm of haemodialysis patients may be essential for ensuring accurate BP monitoring.

## Introduction

Subclavian artery stenosis is characterized by a significant obstruction (stenosis/occlusion) in the proximal vasculature that supplies the upper extremity. This disease is considered a potential marker of diffuse atherosclerosis, and was associated with a risk of cardiovascular mortality.^1^ A bilateral brachial blood pressure measurement with a left–right systolic blood pressure (BP) discrepancy of >15 mmHg is recommended to screen for subclavian artery stenosis.^1^ Subclavian artery stenoses, however, are most often asymptomatic and therefore are underdiagnosed.^1^

Patients undergoing dialysis may carry a potential burden of atherosclerosis, which may be linked to the risk of subclinical subclavian artery stenosis. However, screening for subclavian artery stenosis is further hindered by the relative or absolute contraindication of measuring blood pressure in the shunt brachial artery. Here, we present a case of a dialysis patient who experienced escalating use of an inotropic drug during haemodialysis due to an undiagnosed subclavian artery stenosis.

## Summary figure

**Figure:**
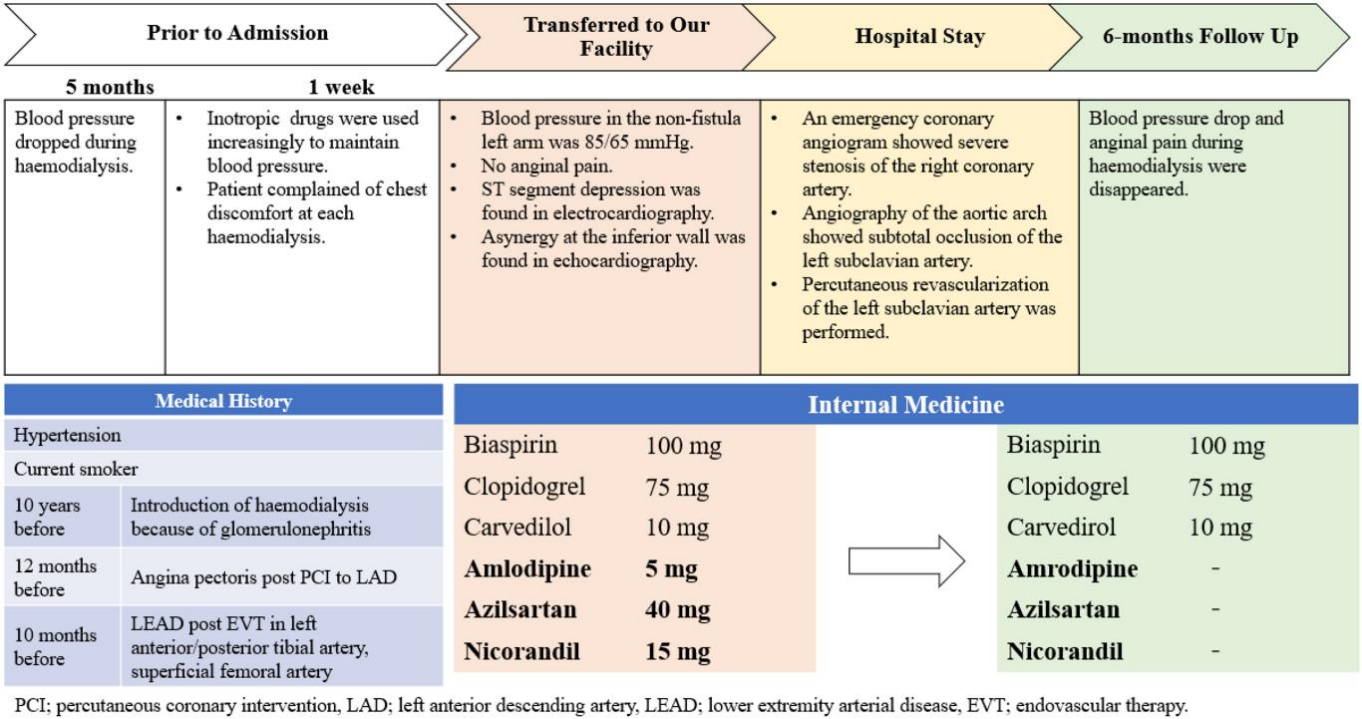


## Case presentation

A 77-year-old woman with end-stage renal disease requiring dialysis presented with a suspected acute coronary syndrome complicated by cardiogenic shock. The patient was a current smoker and had received maintenance dialysis for chronic glomerulonephritis 10 years prior. The patient underwent percutaneous coronary intervention in the left anterior descending artery one year ago, and endovascular therapy (EVT) for severe stenosis of the left anterior tibial artery, post-tibial artery, and superficial femoral artery 10 months ago. Endovascular therapy of the right lower extremity was planned before the patient presented with the current symptoms. She was on dual antiplatelet therapy (i.e. aspirin 100 mg and clopidogrel 75 mg) and antihypertensive medications (i.e. carvedilol 10 mg, amlodipine 5 mg, and azilsartan 40 mg). She managed her activities of daily living without any assistance from family members.

Five months before presentation, BP dropped during dialysis, and for a week, inotropic drugs were used increasingly to maintain BP. She complained of chest discomfort at each haemodialysis session and underwent an electrocardiogram each time, with no abnormal findings. On presentation, the BP in the non-fistula left arm was 85/65 mmHg, with a regular pulse rate of 82 beats/min, and oxygen saturation within the normal range. Exam was pertinent for a heart that was normal rate and regular rhythm without any murmurs. There were neither signs of elevated jugular venous pressure nor lower extremity oedema. Her lungs were clear to auscultation. Electrocardiography showed a pattern of ST segment depression in leads II, III, aVF, and V4 through 6, and echocardiographic findings showed mild hypokinesis at the inferior wall. Cardiac enzyme levels were within normal limits, except for an elevated Troponin-T (Troponin-T 0.12 ng/mL, normal range 0–0.10 ng/mL).

**Figure 1:**
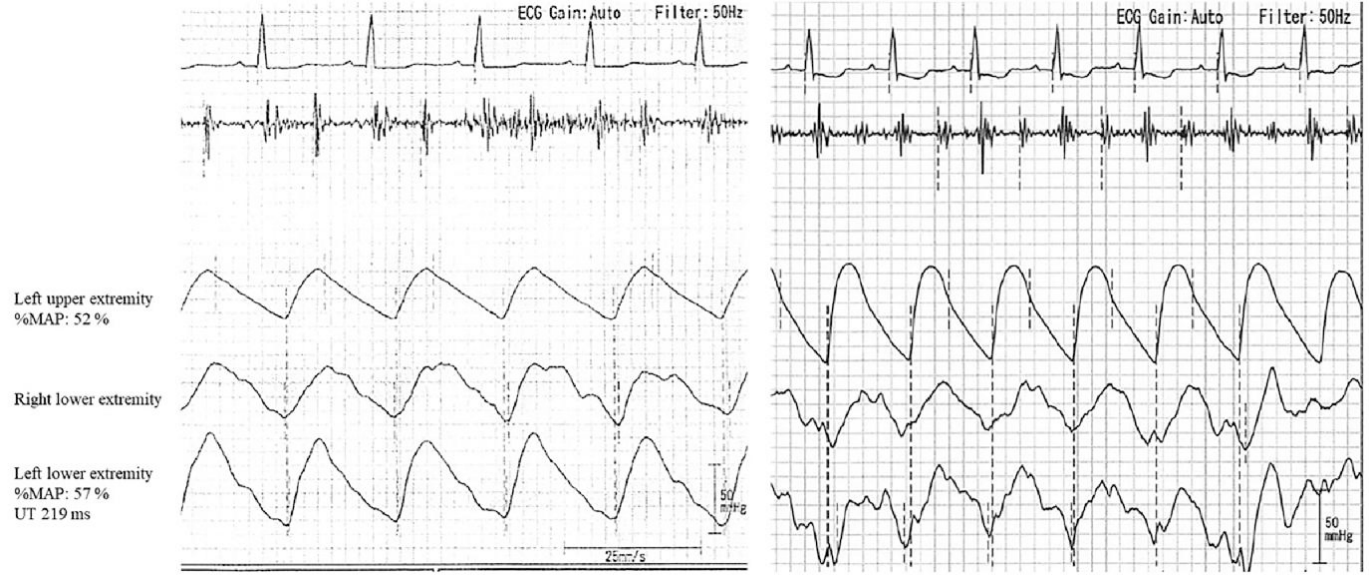
Pressure wave before (left) and after (right) EVT. Left: the pressure waveform on the left upper arm is blunted. Right: after EVT, blunting of the left upper arm pressure waveform in the ankle-brachial index improved. EVT, endovascular therapy; UT, upstroke time; %MAP, %mean artery pressure.

**Figure 2:**
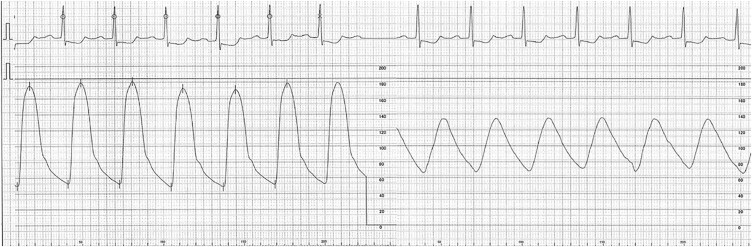
Aortic pressure wave of proximal aortic artery (left) and left brachial artery (right). Catheterization revealed that the blood pressure in the aortic arch was 70 mmHg higher than that in the left brachial artery.

An emergency coronary angiogram of the right coronary artery revealed 99% stenosis, but it was also noted to be hypoplastic. Therefore, the decision was made to proceed with optimal medical therapy. During catheterization, we found that the BP of the aortic arch was 70 mmHg higher than that of the left brachial artery (*Figure 1*). Angiography of the aortic arch showed subtotal occlusion of the left subclavian artery (*Figure 2*).

For accurate BP monitoring during dialysis, EVT was performed on Day 20 after admission. After placement of a 6 Fr sheath in the right common femoral artery, the guidewire was passed through the subtotal occlusion in the left proximal portion of subclavian artery with antegrade crossing, and successful EVT was achieved by SHIDEN HP 5.0 mm × 20 mm (KANEKA®) pre-dilation and Express LD 7.0 mm × 57 mm × 90 cm (Boston Scientific®) angioplasty.

**Figure 3:**
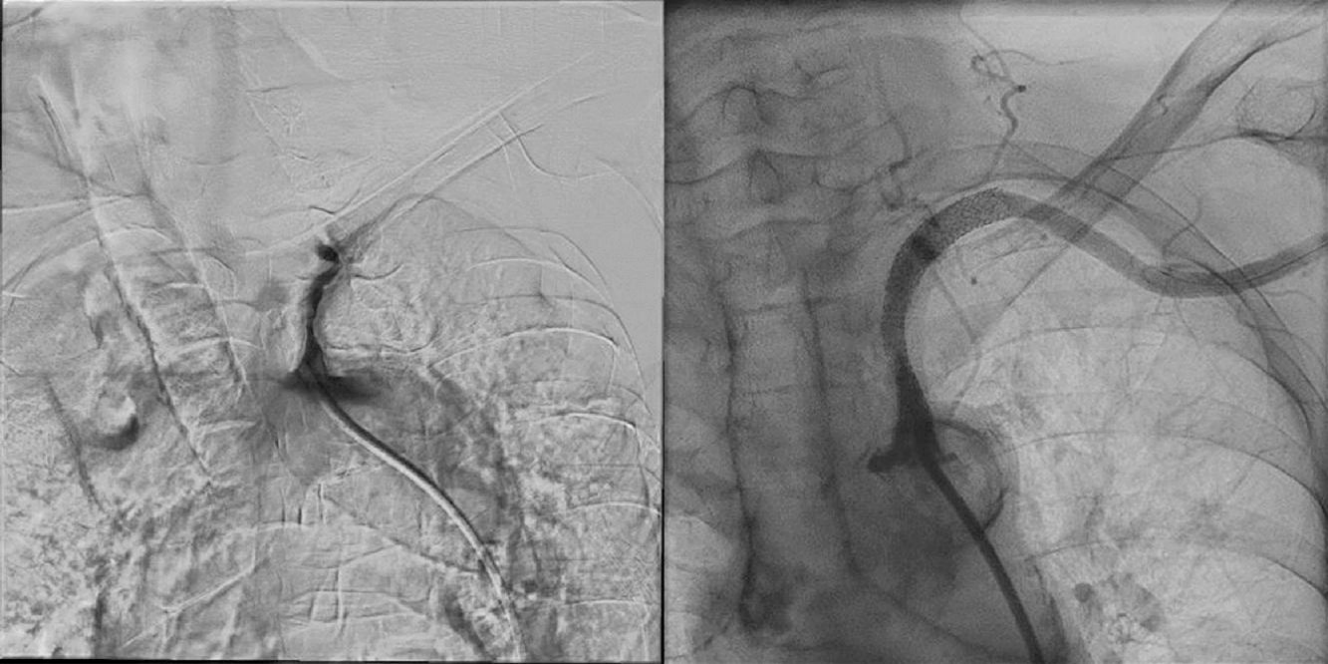
Angiography before (left) and after (right) EVT. Left: digital subtraction angiography showed left subclavian artery subocclusion. Right: after EVT, endovascular treatment showed good flow recovery in the subclavian artery. EVT, endovascular therapy.

Following the intervention, the patient experienced an increase in systolic BP on the left arm, rising from 70 to 120 mmHg. Additionally, the upstroke time in the pulse volume waveform decreased from 240 to 150 ms, as depicted in *Figure 3.* The patient was discharged 6 days after the procedure with neither impairment of her daily activities nor any changes to her medications during hospitalization.

### Follow-up

Six months after being discharged from our hospital, the patient died from septic shock caused by cholecystitis. Until her death, she did not experience chest discomfort or a drop in blood pressure during haemodialysis.

## Discussion

The present case exhibited multiple atherosclerotic comorbidities [i.e. coronary artery disease, lower extremity artery disease (LEAD), and haemodialysis], and was measured with pulse wave analysis. However, the patient’s underlying subclavian artery stenosis was initially undiagnosed over several months, leading to escalating use of an inotropic drug. It was also noted that there was an extreme difference of >70 mmHg between central arterial pressure and peripheral arterial pressure during catheterization. Our case highlights the importance of screening for subclavian artery stenosis in dialysis patients who have high cardiovascular burden.

The prevalence of subclavian artery stenosis has been reported to be associated with old age, hypertension, and LEAD.^1^ Although the high atherosclerotic burden in patients undergoing dialysis has been well established,^2^ the prevalence of subclavian artery stenosis in these patients remains unclear. Therefore, our case may encourage clinicians to screen for the presence of subclavian artery stenosis in patients undergoing dialysis, particularly with old age and/or with comorbidities such as LEAD.

Analysis of pulse volume waveform is recognized as the gold standard for arterial stiffness assessment.^3^ As upstroke time or %mean artery pressure is reported to be useful not only in LEAD diagnosis but also for cardiovascular risk assessment as vascular markers of atherosclerosis,^4,5^ assessing PWV may be an alternative way of screening for subclavian artery stenosis. Our case was assessed using a pulse wave to monitor the severity or treatment response for LEAD, and we observed that the upstroke time on the subclavian artery stenosis arm was prolonged, as previously reported.^6^ These findings suggest that these indices may be clinically relevant for screening the presence of subclavian artery stenosis, even in dialysis patients. Furthermore, continuous-wave Doppler sonography might be an alternative tool for detecting subclavian stenosis or occlusion and reversal of blood flow in the vertebral artery in dialysis patients.^7^

According to the European Society of Cardiology guidelines for vascular surgery, EVT is recommended even in asymptomatic cases of significant bilateral subclavian stenosis or obstruction to ensure accurate BP monitoring.^8^ Haemodialysis patients with unilateral subclavian artery stenosis in the non-fistula arm may encounter similar challenges, as many clinicians are concerned about measuring BP in the fistula arm. Additionally, intradialytic hypotension may have poor prognosis in haemodialysis patients, making blood pressure measurement crucial for monitoring their haemodynamic status.^9,10^ Several reports on EVT for subclavian artery stenosis in dialysis patients focused primarily on stenosis in the shunt arm, particularly in those undergoing coronary artery bypass grafting with steal syndrome.^11–13^ The effectiveness of EVT in the case presented here has been scarcely reported. Although the guidelines do not specifically recommend EVT for this subset of patients, our case highlights the potential benefits of EVT for unilateral subclavian stenosis in the non-fistula arm.

Furthermore, hypotension during haemodialysis was suggested as a surrogate for high mortality in patients on dialysis.^9,10^ Our case was escalated use of an inotropic drug, possibly resulting in chest discomfort due to increased central pressure levels. Therefore, our case suggests that poor prognosis of haemodialysis-induced hypotension might be partly confounded by the presence of subclavian artery stenosis, and a prospective study investigating the prevalence and prognostic value of subclavian artery stenosis in dialysis patients is warranted.

### Strengths and limitations

The presented findings have several potential strengths and limitations. The strength includes the assessment of aortic pressure and angiogram in a dialysis patient without typical stenosis-related symptoms, who had subclavian artery stenosis in the non-fistula arm.

The main limitation was that the patient did not undergo a diagnostic workup despite experiencing chest discomfort 1 week before the haemodialysis session. In cases where chest discomfort is present, especially in patients with cardiovascular burden, an urgent assessment for underlying ischaemic disease should be advisable. However, the timing of diagnosing the exacerbation of ischaemic disease may be limited by several factors, such as symptom severity, findings of electrocardiogram and the absence of a cardiologist.^14,15^

## Conclusion

We encountered a patient requiring haemodialysis who experienced a drop in BP during haemodialysis due to subclavian artery stenosis. This case highlights the importance of screening for subclavian artery stenosis in patients undergoing dialysis. A prospective cohort study is warranted to clarify the prevalence and prognostic value of subclavian artery stenosis in haemodialysis patients.

## Data Availability

The dataset generated during this case presentation is available from the corresponding author on reasonable request.

## Lead author biography

I am a clinician who received my clinical training at Tokyo Medical University Hospital and later practiced as a cardiologist at an affiliated facility of the same hospital.

## Notes

**Consent:** The author confirmed that written consent for submission and publication of this case report including image(s) and associated text has been obtained from the patient in line with COPE guidance.

**Funding:** None.
